# Supplementary material for: Identification of photoperiod-induced specific miRNAs in the adrenal glands of Sunite sheep (Ovis aries)
Source: Front Vet Sci. 2022 Jul 22;9:888207. doi: 10.3389/fvets.2022.888207 (PMC9354845; doi:10.3389/fvets.2022.888207)
Supplement: Supplementary file 1 [file Data_Sheet_1.ZIP › Supplementary file/miRDeep2 file.zip!/miRDeep2 file/Novel_Structure/LP42A3/Novel_32.pdf]

Provisional ID	:	NC_019459.2_3512
Score total	:	2
Score for star read(s)	:	-1.3
Score for read counts	:	0
Score for mfe	:	1.7
Score for randfold	:	1.6
Score for cons. seed	:	
Total read count	:	266
Mature read count	:	266
Loop read count	:	0
Star read count	:	0

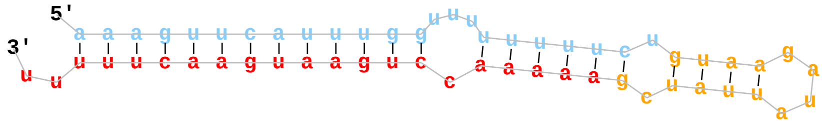

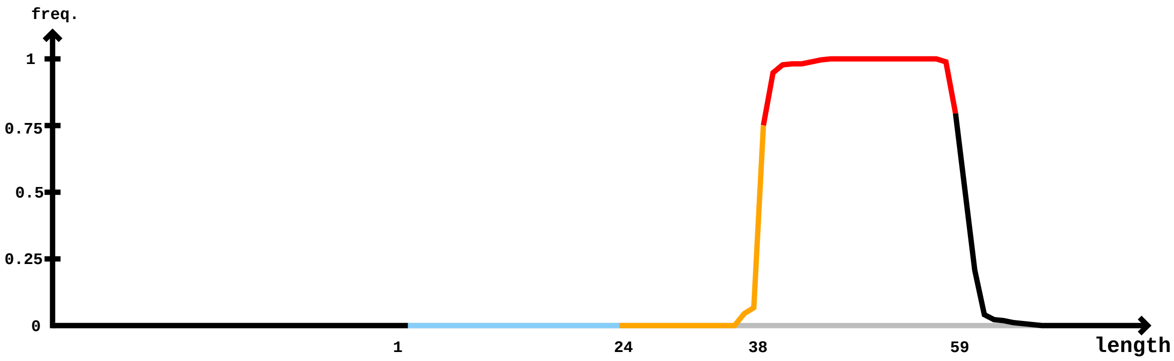

Star Mature

```
5'- cuuaugaaucgucuuucuauuaucagguuggccgaaaaguucauuugguuuuuuuucuguaagauauuaucgaaaaaccugaaugaacuuuuuggccaacucaacccaauac   -3'   exp
    .....(((......)))........(((((((((((((((((((((((...((((((.((((....)))).)))))).))))))))))))))))))))))))...........   reads   mm   sample
    ......................................................................Agaaaaaccugaaugaacuuu.....................   1    1    seq
    ......................................................................cgaaaaaccugaaugaacuuuu....................   1    0    seq
    ......................................................................Agaaaaaccugaaugaacuuuu....................   10   1    seq
    .......................................................................gaaaaaccugaaugaacuuu.....................   1    0    seq
    .......................................................................gaaaaaccugaaugaacuuuu....................   2    0    seq
    .......................................................................gaaaaaccugaaCgaacuuuu....................   1    1    seq
    .......................................................................gaaaaaUcugaaugaacuuuuu...................   1    1    seq
    .......................................................................gaaaaaccugaaugaacCuuuug..................   1    1    seq
    ........................................................................aaaaaccugaaCgaacuuu.....................   1    1    seq
    ........................................................................aaaaaccAgaaugaacuuuu....................   3    1    seq
    ........................................................................aaaaaUcugaaugaacuuuu....................   23   1    seq
    ........................................................................aaaaaccCgaaugaacuuuu....................   2    1    seq
    ........................................................................aaaaaccugaaCgaacuuuu....................   7    1    seq
    ........................................................................aaaaaccAgaaugaacuuuuu...................   2    1    seq
    ........................................................................aaaaaccCgaaugaacuuuuu...................   1    1    seq
    ........................................................................aaaaaccugaaugGacuuuuu...................   1    1    seq
    ........................................................................aaaaaccugaaugaacuuuuu...................   14   0    seq
    ........................................................................aaaaaUcugaaugaacuuuuu...................   65   1    seq
    ........................................................................aaaaaccugaaGgaacuuuuu...................   3    1    seq
    ........................................................................aaaaaccugaaugaacuuuuC...................   1    1    seq
    ........................................................................Gaaaaccugaaugaacuuuuug..................   1    1    seq
    ........................................................................aaaaaUcugaaugaacuuuuug..................   42   1    seq
    ........................................................................aaaaaccugaaugaacuuuuug..................   13   0    seq
    ........................................................................aaaaaccugaaugaGcuuuuug..................   1    1    seq
    ........................................................................aaaaaccugaaugaacuuuuugC.................   3    1    seq
    ........................................................................aaaaaccugaaugaacuuuuugA.................   1    1    seq
    .........................................................................aaaaccugaaugaacuuuu....................   2    0    seq
    .........................................................................aaaCccugaaugaacuuuuu...................   1    1    seq
    .........................................................................aaaaccAgaaugaacuuuuu...................   2    1    seq
    .........................................................................aaaaccCgaaugaacuuuuug..................   6    1    seq
    .........................................................................Gaaaccugaaugaacuuuuug..................   1    1    seq
    .........................................................................aaCaccugaaugaacuuuuug..................   1    1    seq
    .........................................................................aaaaccugaaugaacuuuuugg.................   30   0    seq
    .........................................................................Caaaccugaaugaacuuuuugg.................   1    1    seq
```

Star Mature

cuuaugaaucgucuuucuauuaucagguuggccgaaaaguucauuugguuuuuuuucuguaagauauuaucgaaaaaccugaaugaacuuuuuggccaacucaacccaauac			
....aGaaccugaaugaacuuuuugg.................	1	1	seq
....aaaaccugaaCgaacuuuuugg.................	1	1	seq
....aaaaccugaaugaacuuuuugC.................	2	1	seq
....Uaaaccugaaugaacuuuuugg.................	1	1	seq
....aaCaccugaaugaacuuuuugg.................	1	1	seq
....aaaaUcugaaugaacuuuuuggc................	2	1	seq
....aaaaccugaaugaacuuuuuggGca..............	1	1	seq
....aaaccugaaugaacuuuu....................	1	0	seq
....aaaccAgaaugaacuuuuu...................	1	1	seq
....aaaccugaaugaacuuuuugg.................	3	0	seq
....aaaccugGaugaacuuuuuggc................	2	1	seq
....aaaccugaaugaacuuuuuggA................	1	1	seq
....aaccugaaugaacuuuuugg.................	1	0	seq
....ccugaaugaacuuuuuggcc...............	1	0	seq
....cUugaaugaacuuuuuggcca..............	1	1	seq
....cugaaugaacuuuuuUgccaa.............	1	1	seq
....cugaaugaacuuuuuggccCac............	1	1	seq
....ugaGugaacuuuuuggccaacu...........	1	1	seq
